# Antimigraine Drug Avitriptan Is a Ligand and Agonist of Human Aryl Hydrocarbon Receptor that Induces CYP1A1 in Hepatic and Intestinal Cells

**DOI:** 10.3390/ijms21082799

**Published:** 2020-04-17

**Authors:** Barbora Vyhlídalová, Kristýna Krasulová, Petra Pečinková, Karolína Poulíková, Radim Vrzal, Zdeněk Andrysík, Aneesh Chandran, Sridhar Mani, Zdenek Dvorak

**Affiliations:** 1 Department of Cell Biology and Genetics, Faculty of Science, Palacky University, Slechtitelu 27, 783 71 Olomouc, Czech Republic; vyhlidalovabara@gmail.com (B.V.); kkrasulova@seznam.cz (K.K.); petra.pecinkova@upol.cz (P.P.); karolina.poulikova@gmail.com (K.P.); radim.vrzal@email.cz (R.V.); 2 Department of Pharmacology, University of Colorado School of Medicine, Aurora, CO 80045, USA; zdenek.andrysik@colorado.edu; 3 Department of Medicinal Chemistry, College of Pharmacy, University of Michigan, Ann Arbor, MI 48109-1065, USA; aneeshcna@gmail.com; 4 Department of Genetics and Department of Medicine, Albert Einstein College of Medicine, Bronx, NY 10461, USA

**Keywords:** Aryl Hydrocarbon Receptor, Antimigraine drugs, Triptans, repurposing

## Abstract

The efforts for therapeutic targeting of the aryl hydrocarbon receptor (AhR) have emerged in recent years. We investigated the effects of available antimigraine triptan drugs, having an indole core in their structure, on AhR signaling in human hepatic and intestinal cells. Activation of AhR in reporter gene assays was observed for Avitriptan and to a lesser extent for Donitriptan, while other triptans were very weak or no activators of AhR. Using competitive binding assay and by homology docking, we identified Avitriptan as a low-affinity ligand of AhR. Avitriptan triggered nuclear translocation of AhR and increased binding of AhR in CYP1A1 promotor DNA, as revealed by immune-fluorescence microscopy and chromatin immune-precipitation assay, respectively. Strong induction of *CYP1A1* mRNA was achieved by Avitriptan in wild type but not in AhR-knockout, immortalized human hepatocytes, implying that induction of CYP1A1 is AhR-dependent. Increased levels of *CYP1A1* mRNA by Avitriptan were observed in human colon carcinoma cells LS180 but not in primary cultures of human hepatocytes. Collectively, we show that Avitriptan is a weak ligand and activator of human AhR, which induces the expression of CYP1A1 in a cell-type specific manner. Our data warrant the potential off-label therapeutic application of Avitriptan as an AhR-agonist drug.

## 1. Introduction

The aryl hydrocarbon receptor (AhR) is a transcription factor belonging to the family of basic helix-loop-helix transcription factors. In its inactive form, the AhR resides in the cytosol in complex with chaperone proteins. Ligand binding to the AhR induces dissociation of the protein complex and triggers nuclear translocation of the ligand-receptor complex. Transcriptionally active heterodimer of AhR with AhR nuclear translocator (ARNT) is formed in the nucleus and it binds to specific response elements in the promotors of AhR-target genes. Ligands of AhR comprise a plethora of structurally diverse compounds, including both xenobiotics (e.g., polyaromatic hydrocarbons, polyhalogenated biphenyls, natural phenolics, benzimidazole proton pump inhibitors) and endogenous substances (e.g., intermediary and microbial metabolites of tryptophan, tetrapyrroles, eicosanoids) [1]. The AhR transcriptionally controls a wide array of genes, including those involved in xenobiotic metabolism, immune homeostasis, cell cycle, differentiation and energy metabolism. Hence, the AhR is a critical player in human physiology (e.g., hematopoiesis) [2] and also in many pathophysiological processes such as diabetes, carcinogenesis, inflammation, infection or cardiovascular diseases [3,4,5]. The attempts for therapeutic and preventive targeting of AhR have emerged in recent years [6]. Both natural and synthetic AhR agonists and antagonists are potential drug candidates. An intriguing and respected strategy in current pharmacotherapy is repositioning (off-label use) of a clinically used drug. Indeed, the AhR active drugs, such as tranilast, flutamide or omeprazole, might be effective chemotherapeutics for the treatment of breast and pancreatic cancers [7]. An anti-leprosy Food and Drug Administration (FDA)-approved drug and AhR antagonist clofazimine suppressed multiple myeloma in transgenic mice [8]. Targeting of AhR with antagonists was suggested as a strategy for delaying the relapse during the treatment of melanoma with vemurafenib [9] or for inhibiting constitutive AhR activity in prostate cancer [10]. The use of AhR ligands is not limited to anti-cancer therapy but given the roles of AhR in the intestines and skin, targeting the AhR is challenging also in the treatment of inflammatory bowel disease (IBD) or skin pathologies. Indeed, tranilast is used in the treatment of atopic dermatitis [11]. The drawback with long term use of these compounds is their side-effects and off-target effects, as reported for omeprazole [12]. Thus, there is a perpetual need for the discovery of safer AhR ligands for future therapeutic use. The suitable candidates for off-targeting AhR could be the antimigraine drugs of triptan class, which have an indole core in their structure. The manifold of indole-based compounds were demonstrated as ligands of AhR, including synthetic xenobiotic indoles (e.g., methylindoles and methoxyindoles) [13], dietary indoles (e.g., indole-3-carbinol and diindolylmethane) [14] and microbial catabolites of tryptophan, such as skatole [15], tryptamine, indole-3-acetate [16] and indole [17]. 

In the current study, we examined the effects of clinically used triptans [18], including Sumatriptan, Naratriptan, Rizatriptan, Eletriptan, Zolmitriptan, Almotriptan and Frovatriptan, on transcriptional activity and functions of AhR. We also tested Avitriptan [19] and Donitriptan [20], triptans that were developed but never marketed. Employing the methods of RT-PCR, western blotting, reporter-gene assays, ChIP-assay, radio-ligand binding assay, protein immune-precipitation, in situ immune-fluorescence and in silico docking, we demonstrate that Avitriptan is a weak ligand and agonist of AhR that induces the expression of the AhR-target genes. Our data warrant the potential therapeutic application of Avitriptan as AhR-agonist drug, which is further promoted by the fact that Avitriptan already passed phase I and II of clinical tests.

## 2. Results

### 2.1. Triptans Are Activators of Human AhR

**Figure 1:**
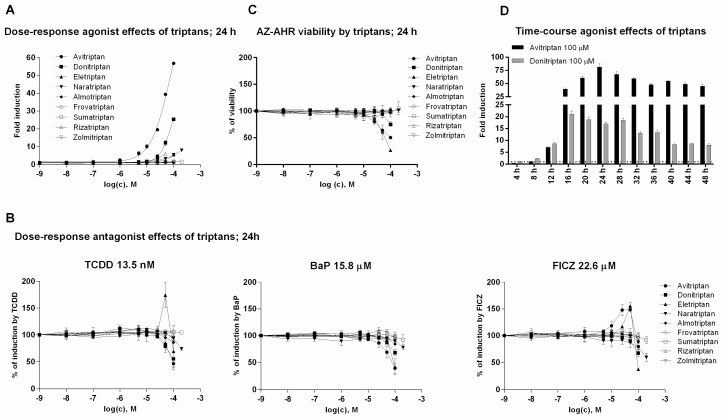
Transcriptional activity of aryl hydrocarbon receptor (AhR). (i) Dose-response analyses–AZ-AHR cells were incubated for 24 h with vehicle (DMSO—dimethyl sulfoxide; 0.1% *v*/*v*) and/or triptans in concentrations ranging from 1 nM to 200 µM, in the absence (agonist mode) or in the presence (antagonist mode) of model AhR agonists comprising TCDD (2,3,7,8-tetrachlorodibenzo-*p*-dioxin; 13.5 nM), BaP (Benzo[a]pyrene; 15.8 µM) and FICZ (6-Formylindolo[3,2-b]carbazole; 22.6 µM). (**A**) Agonist analyses. (**B**) Antagonist analyses. (**C**) MTT cell viability assay. (ii) Time-course analyses—AZ AHR cells were incubated for 0–48 h with DMSO (0.1% *v*/*v*), TCDD (10 nM), Avitriptan (100 µM) and Donitriptan (100 µM) (**D**). Following the treatments cells were lysed and luciferase activity was measured. Experiments were performed in three consecutive passages of AZ-AHR cells. Data are expressed as a fold induction of luciferase activity over control cells (agonist) or as percentage of maximal induction (antagonist) and they are the mean ± SD from measurements performed in quadruplicates.

In the first series of experiments, we examined agonist and antagonist effects of triptans on AhR using reporter gene assay. For this purpose, we incubated stably transfected human hepatoma AZ-AHR cells for 24 h with triptans (maximal tested concentrations were selected based on limited solubility of individual compounds) in the presence or the absence of diverse AhR agonists, including TCDD (2,3,7,8-tetrachlorodibenzo-*p*-dioxin), BaP (Benzo[a]pyrene) and FICZ (6-Formylindolo[3,2-b]carbazole). Avitriptan, Donitriptan and Naratriptan activated dose-dependently AhR, while other triptans were inactive (Figure 1A). The activation was rather weak and the relative efficacy of triptans in 100 µM concentration as compared to TCDD (luciferase induction approx. 1900-fold) decreased in order—Avitriptan (~3%) > Donitriptan (~1.5%) > Naratriptan (~0.5%). Tested triptans did not display antagonist effects against AhR (Figure 1B) and the decrease of agonists-induced luciferase activity of AhR by several triptans were rather due to their intrinsic cytotoxicity in AZ-AHR cells (Figure 1C). Time-course analyzes revealed the differential dynamics of AhR time-dependent activation by Avitriptan and Donitriptan, which were selected as two most active triptans for detailed investigation. In short periods of incubation (<12 h), Donitriptan was a more robust activator than Avitriptan. In comparison, we observed after prolonged incubation (>12 h) (Figure 1D), which is consistent with dose-response inverse effects after 24 h (Figure 1A). The plausible explanation for such a behavior could be existing substantial differences between the degrees of triptans interactions with drug transporters [21].

### 2.2. Avitriptan and Donitriptan induce CYP1A1 in Hepatic and Intestinal Cells via AhR

**Figure 2:**
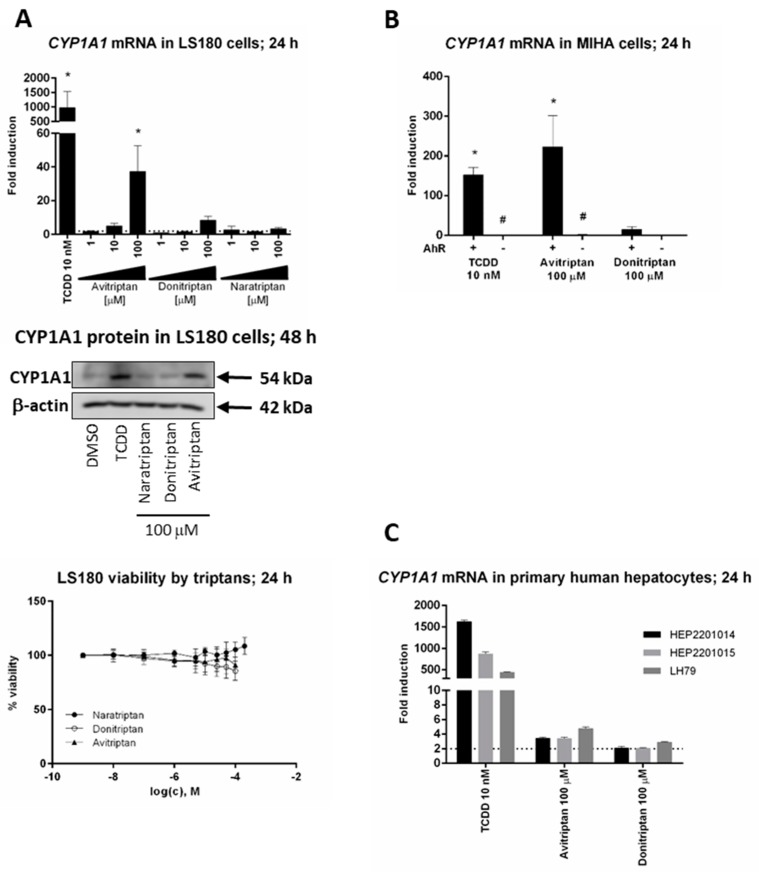
Induction of CYP1A1. Cells were incubated with triptans (100 µM), TCDD (10 nM) and/or vehicle (0.1% DMSO) for 24 h (mRNA analyses, MTT test) and 48 h (protein analyses). The levels of *CYP1A1* mRNA and protein were determined by the means of RT-PCR and western blot, respectively. (**A**) Experiments in three consecutive passages of human colon adenocarcinoma cells LS180. Upper bar graph shows a fold induction of *CYP1A1* mRNA over control cells. Data are expressed as mean ± SD. RT-PCR was carried out in triplicates (technical replicates). * = significantly different from DMSO-treated cells (*p* < 0.05); dashed horizontal insert shows borderline 2-fold induction. Representative western blot of CYP1A1 protein is shown. Bottom plot shows MTT cell viability assay. (**B**) Human immortalized hepatocytes MIHA-(AhR^+/+^) and MIHA-(AhR^−/−^). Bar graph shows a fold induction of *CYP1A1* mRNA over control cell. Data are expressed as mean ± SD from three consecutive cell passages. RT-PCR was carried out in triplicates (technical replicates). *= significantly different from DMSO-treated cells (*p* < 0.05); #= significantly different from wild-type cells (*p* < 0.05) (**C**) Experiments in primary human hepatocytes cultures obtained from three different liver tissue donors. Bar graph shows a fold induction of *CYP1A1* mRNA over control cells. Data are expressed as mean ± SD. RT-PCR was carried out in triplicates (technical replicates).

We studied the induction of prototypical AhR target gene CYP1A1 by triptans in hepatic and intestinal cell models. Avitriptan and Donitriptan but not Naratriptan, dose-dependently induced *CYP1A1* mRNA in intestinal adenocarcinoma cells LS180 after 24 h of incubation (Figure 2A). The induction was rather weak and the levels of *CYP1A1* mRNA were increased approx. 38-fold and 8-fold by Avitriptan and Donitriptan in 100 µM concentrations, respectively. The relative efficacies of Avitriptan (~4%) and Donitriptan (~1%) were consistent with those observed in reporter gene assays in AZ-AHR cells. The level of CYP1A1 protein in LS180 cells after 48 h of incubation was significantly increased only by Avitriptan (Figure 2A). Importantly, unlike in hepatoma AZ-AHR cells, Avitriptan and Donitriptan were not cytotoxic in intestinal LS180 cells (Figure 2A). Induction of *CYP1A1* mRNA in immortalized human hepatocytes MIHA, incubated for 24 h with TCDD, Avitriptan and Donitriptan was 150-fold, 215-fold and 16-fold, respectively. Triptans did not induce *CYP1A1* mRNA in AhR knockout variant of MIHA cells, implying the AhR-dependent induction of CYP1A1 by triptans (Figure 2B). In contrast, in typical primary human hepatocytes cultures, prepared from healthy liver tissue donors, Avitriptan and Donitriptan caused an only weak and non-significant increase of *CYP1A1* mRNA, by 2-fold and 4-fold respectively, while TCDD induced *CYP1A1* mRNA between 400-fold and 1600-fold (Figure 2C). Cell type-specific induction of CYP1A1 could be due to the extensive oxidative metabolism, which was described for Avitriptan [22,23]. 

### 2.3. Avitriptan Is a Low-Affinity Ligand of AhR

**Figure 3:**
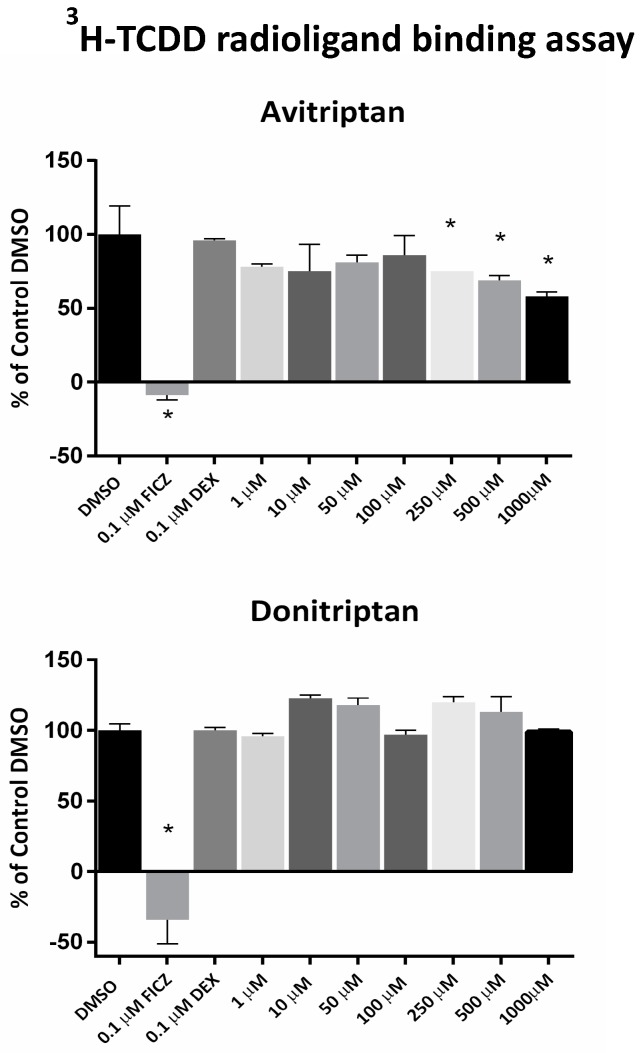
Radio-ligand binding assay. Cytosolic protein from Hepa1c1c7 cells was incubated with Avitriptan (1–1000 µM), Donitriptan (1–1000 µM), FICZ (10 nM; positive control), dexamethasone (100 nM; negative control) or vehicle (DMSO; 0.1% *v*/*v*; corresponds to specific binding of [^3^H]-TCDD = 100%) in the presence of 2 nM [^3^H]-TCDD. Specific binding of [^3^H]-TCDD was determined as a difference between total and non-specific TCDF (200 nM; 2,3,7,8-tetrachlorodibenzofuran) reactions. * = significantly different from negative control (*p* < 0.05). Three independent experiments were performed, and the incubations and measurements were done in triplicates in each experiment (technical replicates). The error bars represent the mean ± SD.

**Figure 4:**
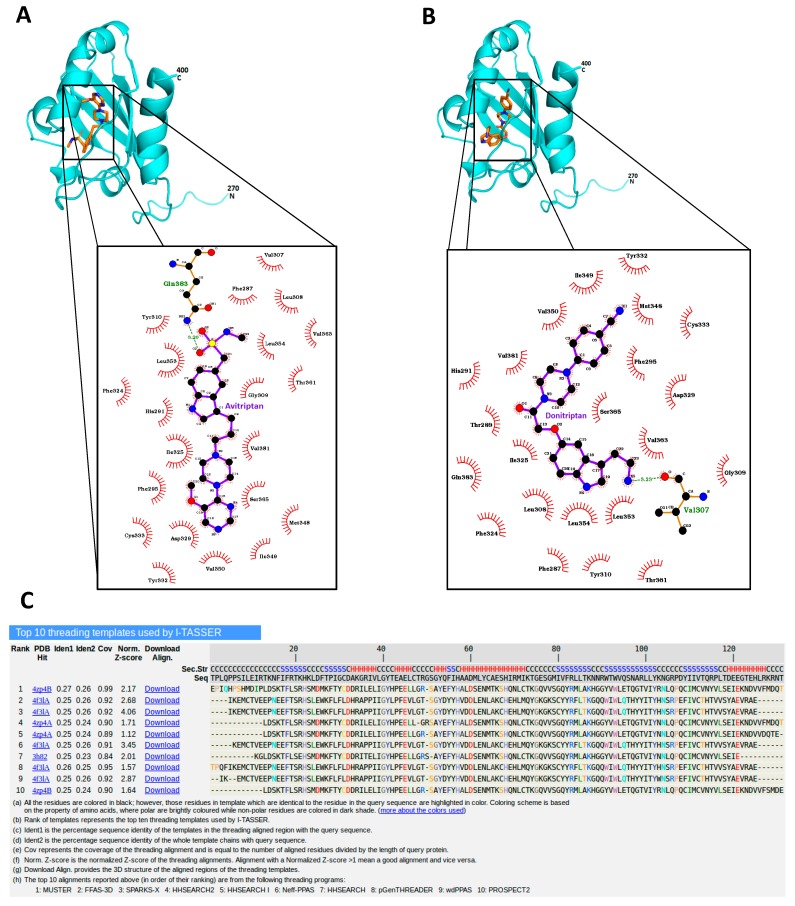
Avitriptan and Donitripan binding at hAhR. Mode of interaction of Avitriptan (**A**) and Donitriptan (**B**) with ligand binding domain of hAhR. Dotted lines denote the hydrogen bonding interaction and the protein residues involved in hydrophobic interactions are shown by red spikes. H-bond distance is shown alongside. (**C**) Top 10 protein templates used by I-TASSER for homology modelling hAhR LBD. The sequence alignment of hAhR LBD versus the templates used in the model building is presented.

Avitriptan and Donitriptan activated AhR and induced the CYP1A1 gene by the AhR-dependent mechanism in multiple cell models. Therefore, we carried out radio-ligand competitive binding assay to determine whether these two triptans interact with AhR directly. Binding of ^3^H-TCDD at mouse AhR was dose-dependently inhibited by Avitriptan, implying that it binds AhR directly. The effects of Avitriptan were weak, suggesting that it is a low-affinity ligand of AhR (Figure 3). While Donitriptan did not displace ^3^H-TCDD from AhR, it is probably very low-affinity ligand of AhR, not detectable by our assay, given the structural and functional similarity with Avitriptan. Corroborating these observations, docking studies also suggested the low-affinity binding of Avitriptan and Donitriptan to human AhR. Both Avitriptan and Donitriptan showed a comparatively similar binding affinity of −3.1 kcal/mol and −3.4 kcal/mol, respectively. Though hydrophobic interactions largely contribute to the binding mode of the compound, both Avitriptan and Donitriptan also form hydrogen bond interactions with the protein backbone N-H or C=O groups (Figure 4). 

### 2.4. Avitriptan and Donitriptan Trigger Nuclear Translocation of AhR

**Figure 5:**
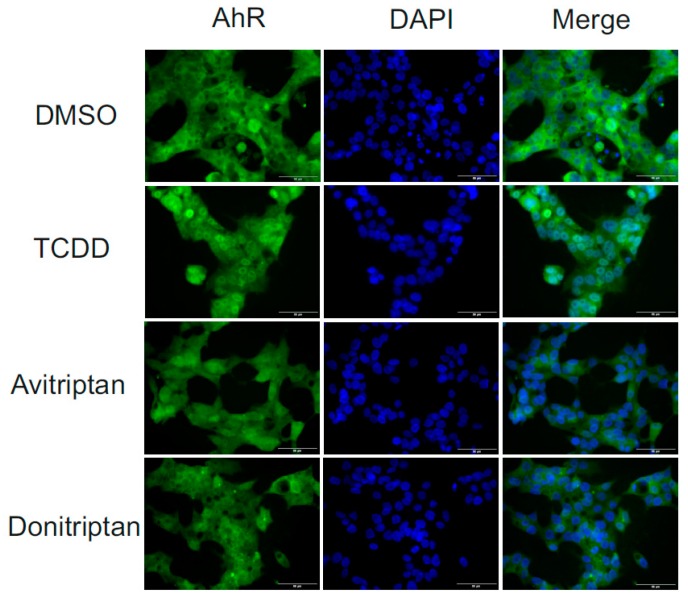
Nuclear translocation of AhR. Fluorescence images depict sub-cellular localization of AhR in LS174T cells incubated for 90 min with vehicle (DMSO; 0.1% *v*/*v*), TCDD (10 nM), Avitriptan (100 μM) and Donitriptan (100 μM). The staining procedure is described in detail in Materials and Methods section. The whole staining protocol was performed in three consecutive cell passages with all tested compounds in duplication. Representative micrographs LS174T cells are shown. Size bars inserted in individual pictures are equal to 50 μM.

Following the binding of the ligand at AhR, the early cellular response is a translocation of AhR from the cytosol to the nucleus. Therefore, we analyzed the nuclear translocation of AhR under the influence of Avitriptan and Donitriptan. We incubated human intestinal LS174T cells for 90 min with the vehicle, TCDD (10 nM), Avitriptan (100 µM) and Donitriptan (100 µM) and we evaluated intracellular localization of AhR using immune-fluorescence. In vehicle-treated cells, AhR was localized predominantly in cytosol (2–9% of positive nuclei), whereas TCDD triggered translocation of AhR into the nucleus (48–63% of positive nuclei). Both Avitriptan and Donitriptan caused nuclear translocation of AhR; however, AhR partially resided in cytosol, which compromised the quantification. Nevertheless, these observations imply weak agonist effects of triptans at AhR (Figure 5).

### 2.5. Formation of AhR-ARNT Heterodimer by Avitriptan and Donitriptan

**Figure 6:**
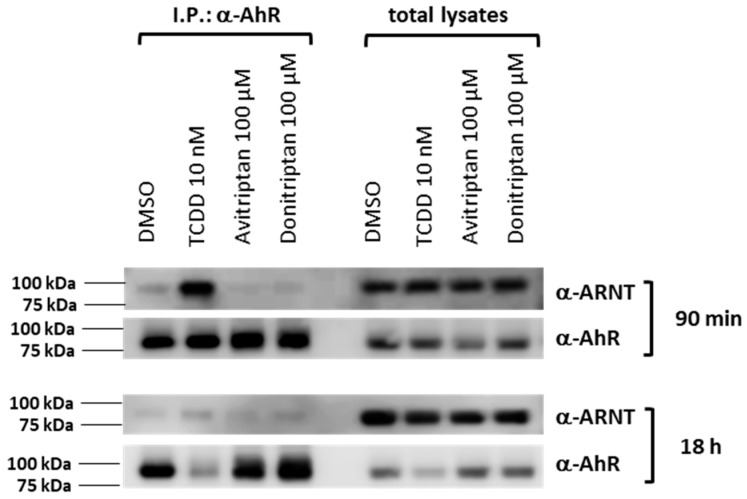
Heterodimerization of AhR with ARNT. Protein immunoprecipitation—formation of AhR-ARNT heterodimer in LS180 cells incubated for 90 min and 18 h with vehicle (DMSO; 0.1% *v*/*v*), TCDD (10 nM), Avitriptan (100 μM) and Donitriptan (100 μM). Representative immunoblots of immuno-precipitated protein eluates and total cell lysates are shown. Experiments were performed in three consecutive cell passages.

Within the canonical AhR signaling pathway, the AhR forms a heterodimer with ARNT upon AhR translocation in the cell nucleus. Thus, we studied the formation of the AhR-ARNT complex by means of protein immune-precipitation in human intestinal LS180 cells incubated for 90 min and 18 h with the vehicle, TCDD, Avitriptan (100 µM) and Donitriptan (100 µM). Robust formation of AhR-ARNT dimer was induced by TCDD but not by Avitriptan and Donitriptan, in cells incubated for 90 min (Figure 6). After 18 h of incubation with TCDD, heterodimerization of AhR with ARNT was very weak, due to the drop in AhR protein levels caused by ligand-dependent AhR degradation, which is also evident by the drastic decrease of AhR protein in total cell lysates. Similarly, faint levels of ARNT protein after co-IP were observed with Avitriptan and Donitriptan (Figure 6). Taking in account the marginal effects of triptans after 90 min of incubation with the degradation of AhR protein in prolonged incubation times, the effects of triptans on AhR-ARNT heterodimerization could not be reliably assessed.

### 2.6. Avitriptan and Donitriptan Enhance the Recruitment of AhR into CYP1A1 Promotor

**Figure 7:**
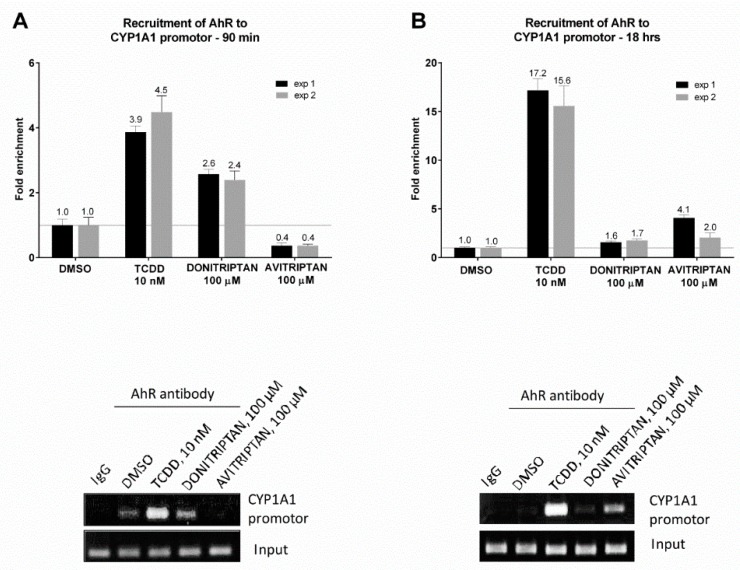
Chromatin immunoprecipitation ChIP–binding of AhR in CYP1A1 promotor. LS174T cells were incubated with Donitriptan (100 µM), Avitriptan (100 µM), TCDD (10 nM) and/or vehicle (0.1% DMSO) for 90 min and 18 h. Bar graphs show enrichment of CYP1A1 promotor with AhR as compared to vehicle-treated cells. A representative DNA fragments amplified by PCR analyzed on a 2% agarose gel are shown. Experiments were performed in two consecutive cell passages.

The capability of Avitriptan and Donitriptan to enhance the binding of AhR in the promotor of its target gene CYP1A1 was studied by ChIP. For this purpose, human intestinal LS174T cells were incubated for 90 min and 18 h with the vehicle, TCDD, Avitriptan (100 µM) and Donitriptan (100 µM). The enrichment of the CYP1A1 promotor with AhR in cells incubated with TCDD for 90 min and 18 h (two consecutive cell passages of LS174T cells) was approx. 4-fold and 16-fold, respectively. Donitriptan increased the binding of AhR in the CYP1A1 promotor approx. 2.5-fold after 90 min of incubation and unlike in the case of TCDD, the effect remained after 18 h of incubation with 1.7-fold induction. On the contrary, Avitriptan caused a weak decrease (0.4-fold) of AhR binding in the CYP1A1 promotor after a short period of incubation (90 min), while prolonged incubation for 18 h yielded approx. 3-fold increased binding (Figure 7). This time-dependent differential dynamics of Avitriptan and Donitriptan at AhR binding to CYP1A1 promoter was consistent with the effects observed in reporter gene assay (Figure 1D) and protein immune-precipitation (Figure 6).

## 3. Discussion

In the current study, we examined the effects of antimigraine drugs of triptan class on AhR-CYP1A1 signaling in human in vitro cell models. Of nine tested triptans, we identified Avitriptan as a lead AhR-active compound. We demonstrate that Avitriptan is a weak agonist and low affinity ligand of AhR, which triggers AhR signaling pathway and induces the expression of AhR target gene CYP1A1 in hepatic and intestinal cells.

The AhR is a Janus-faced actor in human physiology and pathophysiology. In the context of intestinal health and disease, the proper activation of the AhR by endogenous, microbial or dietary ligands has beneficial and protective roles in the onset and progression of IBD and other intestinal pathologies. Consistently, insufficient endogenous activation of the AhR, due to the microbiome dysregulation or for dietary reasons, is the risk factor for onset and progression of IBD [4,5,24,25]. Excessive activation of the AhR by xenobiotic ligands such as environmental pollutants or drugs is also detrimental for intestinal health. The key for dual roles of the AhR in intestinal health and disease is not entirely elucidated. Ligand-dependent activation of the AhR can result in an extremely diverse spectrum of biological and toxic effects that occur in a ligand-, species- and tissue-specific manner [26]. On the basis of a novel computational approach for molecular docking to the homology model of the AhR LBD, specific residues within the AhR binding cavity that play a critical role in binding of three distinct groups of chemicals were recently predicted and experimentally confirmed by Giani Tagliabue et al. [27]. A ligand-selective structural hierarchy controlling dimerization of the AhR with ARNT and the recognition of target DNA was described by Seok et al. [28]. Due to its broad roles, not limited to the intestinal health, the AhR is an emerging therapeutic target for the pharmacotherapy of several diseases, including atopic dermatitis, intestinal inflammation or cancer [7,11,29].

Avitriptan was developed by Bristol-Myers Squibb [30] and reached phase III clinical trials. However, it was suspended because, in high doses (150 mg), transiently elevated liver enzymes were reported [31]. Avitriptan is rapidly absorbed from the small intestine and the speed of absorption of cMAX but not AUC, differs between fed and fasted subjects [32]. Plasma maximum concentrations of Avitriptan (cMAX) following oral administration reached up to ~ two (2) µM [19,22]. Intravenous application of Avitriptan (10 mg) resulted in cMAX of ~ 1 µM [22,33]. The overall bioavailability of orally administered Avitriptan is 17% [19,22,34]. Taken together, the low oral bioavailability of Avitriptan and consequently, its low plasma levels are desirable features if considering Avitriptan orally as an off-label drug for the local therapy of IBD. Also, a recent estimate based on recommended dose and published a fecal excreted fraction of 200 marketed drugs, reports globally >100-times higher drug concentrations in the gut as compared to blood [35]. It implies that oral administration of Avitriptan would result in intra-intestinal local concentrations sufficiently high to activate AhR (≈100 µM), while systemic blood levels will be kept bellow two (2) µM.

We observed cell-specific induction of AhR target gene CYP1A1 in hepatic and intestinal cells. We may only speculate about the mechanisms underlying cell-specific induction, which may comprise differential cellular uptake/intake, metabolism of Avitriptan or distinct interactions with the AhR signaling pathway in cancer (LS180), immortalized (MIHA) and normal cells (primary human hepatocytes). Nevertheless, the lack of CYP1A1 induction in primary cultures of normal human hepatocytes may be considered favorable, in terms of no AhR systemic effects of orally Avitriptan intended for local intestinal treatments.

Based on the data reported in the current study, we propose the possibility to repurpose (off-target use) formerly anti-migraine Avitriptan for local intestinal use as anti-IBD treatment through the AhR. This is supported by the facts that—(i) The AhR is emerging and suitable therapeutic target in IBD; (ii) Avitriptan is a ligand and agonist of the AhR; (iii) Avitriptan passed phase I and phase II of clinical studies, which may accelerate its introduction in clinical use; (iv) Orally Avitriptan has low bioavailability and it is not toxic to intestinal cells, which favors its local use in IBD treatment without having undesirable systemic effects. 

In conclusion, our data reporting activation of AhR by Avitriptan warrant potential off-label therapeutic application of Avitriptan as a AhR-agonist drug in the treatment of intestinal inflammatory pathologies. Ongoing studies should focus on in vitro and in vivo anti-inflammatory capability of Avitriptan.

## 4. Materials and Methods

### 4.1. Chemicals

Almotriptan malate (purity ≥ 98%; cat# SML1210), Avitriptan fumarate (purity ≥ 98%; cat# BM0009), Donitriptan monohydrochloride (purity ≥ 98%; cat# D9071), Eletriptan hydrobromide (purity ≥ 98%; cat# PZ0011), Frovatriptan succinate monohydrate (purity ≥ 97%; cat# SML1291), Zolmitriptan (purity ≥ 98%; cat# SML0248), Benzo[a]pyrene (BaP; B1760, Lot SLBS0038V, purity 99%), 5,11-Dihydro-indolo[3,2-b]carbazole-6-carboxaldehyde, 6-Formylindolo[3,2-b]carbazole (FICZ; SML1489, Lot 0000026018, purity 99.5%), dimethylsulfoxide (DMSO), Triton X-100, bovine serum albumin and hygromycin B were purchased from Sigma-Aldrich (Prague, Czech Republic). Naratriptan hydrochloride (purity 95%; cat# SC-212362), Rizatriptan benzoate (purity 99%; cat# SC-219983), Sumatriptan (purity 98%; cat# SC-473020) were from Santa Cruz Biotechnology (Santa Cruz, CA, USA). 2,3,7,8-tetrachlorodibenzo-*p*-dioxin (TCDD) was from Ultra Scientific (Rhode Island, USA). 2,3,7,8-tetrachlorodibenzofuran (TCDF) was from Ambinter (Orleáns, France). Luciferase lysis buffer was from Promega (Madison, California, USA). DAPI (4′,6-diamino-2-phenylindole) was from Serva (Heidelberg, Germany). [^3^H]-TCDD (purity 98.6%; ART 1642, Lot 181018) was purchased from American Radiolabeled Chemicals. Bio-Gel^®^ HTP Hydroxyapatite (1300420, Lot 64079675) was obtained from Bio-Rad Laboratories. All other chemicals were of the highest quality commercially available. Chemical structures of tested triptans are depicted in Figure S1.

### 4.2. Cell Cultures

Human Caucasian colon adenocarcinoma cells LS180 (#87021202) and LS174T (#87060401) and mouse hepatoma Hepa1c1c7 cells (#95090613) were purchased from the European Collection of Cell Cultures (ECACC) and used in passage number 5C12. Stably transfected gene reporter cell line AZ-AHR was described elsewhere [36]. Cells were maintained at 37 °C and 5% CO_2_ in a humidified incubator. Primary human hepatocytes cultures HEP2201014 (male, 76 years) and HEP2201015 (male, 72 years) were purchased from Biopredic International (Rennes, France). Human hepatocytes culture LH79 (male, 60 years) was prepared at the Faculty of Medicine, Palacky University Olomouc. Liver tissue was obtained from Faculty Hospital Olomouc, Czech Republic and the tissue acquisition protocol followed the requirements issued by “Ethical Committee of the Faculty Hospital Olomouc, Czech Republic” and Transplantation law #285/2002 Coll. Primary human hepatocyte cultures were maintained in serum-free cultivation medium.

Immortalized non-tumorigenic human hepatocyte cell line MIHA was a generous gift from Dr. Xia Wang and Dr. Jayanta Roy-Chowdhury (Albert Einstein College of Medicine, Yeshiva University, NY, USA). AhR knock-out (AhR^−/−^) and control clones (AhR^+/+^) were constructed as follows—Parental line was transiently transfected with a mix of pSpCas9(BB)-2A-GFP (PX458) plasmids encoding two gRNAs (AAGTCGGTCTCTATGCCGCT and AGACCGACTTAATACAGAGT) targeting second exon of the AhR gene. Single cell clones were sub-cultured and successful knock-out was confirmed by western blot.

### 4.3. Cytotoxicity Assay

Cells were incubated for 24 h with tested compounds, vehicle (DMSO; 0.1% *v*/*v*) and Triton X-100 (1%, *v*/*v*), using multi-well culture plates of 96 wells. MTT test was performed and absorbance was measured spectrophotometrically at 540 nm on Infinite M200 (Schoeller Instruments, Prague, Czech Republic). The data were expressed as the percentage of cell viability, where 100% and 0% represent the treatments with vehicle and Triton X-100, respectively.

### 4.4. Reporter Gene Assay

The stably transfected human hepatoma gene reporter cells AZ-AHR [36] were seeded at 96-well culture plates and incubated with test compounds as indicated in detail in figure legends. Thereafter, the cells were lysed, and luciferase activity was measured on a Tecan Infinite M200 Pro plate reader (Schoeller Instruments, Czech Republic). Measurements were carried out in quadruplicates (technical replicates).

### 4.5. Isolation of RNA and qRT-PCR

The total RNA was isolated by TRI Reagent^®^ (Sigma-Aldrich, St. Louis, MO, USA) and cDNA was synthesized using M-MuLV Reverse Transcriptase (New England Biolabs, Ipswich, MA, USA) in the presence of random hexamers (New England Biolabs, USA). The levels of *CYP1A1* and *glyceraldehyde-3-phosphate dehydrogenase* [GAPDH] mRNAs were determined using the Light Cycler^®^ 480 II apparatus (Roche Diagnostic Corporation, Prague, Czech Republic), as described elsewhere [37]. Measurements were carried out in triplicates. Gene expression was normalized to *GAPDH* as a housekeeping gene. The data were processed by the delta-delta method.

### 4.6. Western Blotting

Total protein extracts were prepared by using ice-cold lysis buffer (150 mM NaCl; 50 mM HEPES; 5 mM EDTA; 1% (*v*/*v*) Triton X-100; anti-protease cocktail, anti-phosphatase cocktail). Protein concentration was determined using Bradford reagent. The amount of protein was adjusted to 25 µg per sample. Samples were separated at standard SDS-PAGE followed by western blotting. The following primary antibodies were used for the detection of target proteins—CYP1A1 (mouse-monoclonal, sc-393979, A-9, dilution 1:500, Santa Cruz Biotechnology) and β-actin (mouse-monoclonal, sc-47778, C4, dilution 1:2000, Cell Signaling Technology). Chemiluminescent detection was performed using horseradish peroxidase-conjugated secondary antibodies (anti-mouse, 7076S, dilution 1:2000, Cell Signaling Technology) and WesternSure^®^ PREMIUM Chemiluminescent Substrate (LI-COR Biotechnology) by C-DiGit^®^ Blot Scanner (LI-COR Biotechnology). Experiments were performed in three consecutive cell passages.

### 4.7. Nuclear Translocation of AhR–Immune Histochemistry

LS174T cells were seeded on chamber slides (ibidi GmbH, Grafelfing, Germany) and cultured for two days. Then, cells were incubated for 90 min with vehicle (DMSO; 0.1% *v*/*v*), TCDD (10 nM), Avitriptan (100 μM) and Donitriptan (100 μM). After the treatment, cells were washed by PBS, fixed with 4% formaldehyde, permeabilized using 0.1% Triton X-100, blocked with 3% bovine serum albumin and incubated with Alexa Fluor 488 labelled primary antibody against AhR (sc-133088, Santa Cruz Biotechnology, USA), as described previously [13]. Nuclei were stained by 4′,6-diamino-2-phenylindole (DAPI) and cells were enclosed by VectaShield^®^ Antifade Mounting Medium (Vector Laboratories Inc., Burlingame, CA, USA). AhR translocation into the nucleus was visualized and evaluated using fluorescence microscope IX73 (Olympus, Japan). The whole staining protocol was performed in three independent experiments with all tested compounds in duplication. The AhR translocation was evaluated visually depending on the distinct signal intensity of AhR antibody in the nucleus and cytosol.

### 4.8. Chromatin Immunoprecipitation (ChIP)

The assay was performed as per the manufacturer recommendations for SimpleChIP Plus Enzymatic Chromatin IP kit (Magnetic Beads) (Cell Signaling Technology; #9005), with minor modifications, as recently described [13]. Briefly, LS174T cells were seeded in a 60-mm dish and the following day they were incubated with Donitriptan (100 µM), Avitriptan (100 µM), TCDD (10 nM) and/or vehicle (0.1% DMSO) 90 min and 18 h at 37 °C. Anti-AhR rabbit monoclonal antibody was from Cell Signaling Technology (D5S6H; #83200). Experiments were performed in two consecutive cell passages.

### 4.9. Radio-Ligand Binding Assay

Cytosolic protein from murine hepatoma Hepa1c1c7 cells (2 mg/mL) was incubated for 2 h at room temperature in the presence of 2 nM [^3^H]-TCDD with Avitriptan (1–1000 µM), Donitriptan (1–1000 µM), FICZ (10 nM; positive control), dexamethasone (100 nM; negative control) or vehicle (DMSO; 0.1% *v*/*v*; corresponds to *specific binding of [^3^H]-TCDD = 100%*). Ligand binding to the cytosolic proteins was determined by the hydroxyapatite binding protocol and scintillation counting. Specific binding of [^3^H]-TCDD was determined as a difference between total and non-specific (TCDF; 200 nM) reactions. Three independent experiments were performed, and the incubations and measurements were done in triplicates in each experiment (technical replicates).

### 4.10. Protein Immune-Precipitation

Formation of AhR-ARNT heterodimer was studied in cell lysates from LS180 cells, which were incubated with TCDD (10 nM), Avitriptan (100 µM), Donitriptan (100 µM) and vehicle (DMSO; 0.1% *v*/*v*) for 90 min and 18 h at 37 °C. Pierce™ Co-Immunoprecipitation Kit (Thermo Fisher Scientific), applying covalently coupled AhR antibody (mouse monoclonal, sc-133088, A-3, Santa Cruz Biotechnology) was used. Eluted protein complexes, in parallel with parental total lysates, were resolved on SDS-PAGE gels followed by Western blot and immuno-detection with ARNT 1 antibody (mouse monoclonal, sc-17812, G-3, Santa Cruz Biotechnology). Chemiluminescent detection was performed using horseradish peroxidase-conjugated anti-mouse secondary antibody (7076S, Cell Signaling Technology) and WesternSure^®^ PREMIUM Chemiluminescent Substrate (LI-COR Biotechnology) by C-DiGit^®^ Blot Scanner (LI-COR Biotechnology).

### 4.11. Molecular Docking Studies

Homology model of the ligand binding domain (LBD) of hAhR was generated using the I-TASSER server [38] based on multiple template structures. Amino acid sequence of hAhR LBD residues 270 C400 was obtained from the UniProt database (UniProt ID: P35869). I-TASSER (Iterative Threading ASSEmbly Refinement), which ranked as one of the best server for protein structure prediction in the recent community-wide Critical Assessment of Techniques for Protein Structure Prediction (CASP), uses a hierarchical approach for protein structure prediction that combines multiple threading, ab initio folding and structure refinement for constructing reliable homology based models. The sequence alignment of top 10 templates used by I-TASSER to homology model of the hAhR LBD is illustrated in Figure 4C. In accordance with previous modelling approaches [13,27], I-TASSER also identified PAS structures as the best template for hAhR modelling, with about 50% of sequence similarity. Out of the five models generated by I-TASSER, the model with the highest C-Score (−0.05) was selected for further study. The chosen hAhR LBD structure was further refined by molecular dynamics simulations using GROMACS v2018.1 simulation package (www.gromacs.org). The model was energy minimized and subjected to 10 ns of molecular dynamics simulation at 298 K and the resultant final structure was subsequently used for docking studies.

Molecular docking of Avitriptan and Donitriptan to the hAhR LBD was performed with Autodock Vina [39]. The structures of the ligands were downloaded from PubChem and then prepared with the AutoDockTools. Site-directed mutagenesis studies in the past have identified that residues Thr283, His285, Phe289, Phe318, Met342, Phe345, Leu347, Ser359 and Gln377 of mouse AhR were involved in binding interactions with ligands [40,41]. In the present study, we used this binding information to derive the docking site in human counterpart of AhR. Accordingly, a docking space of 20 × 17 × 22 Å size centered on the pocket lined by residues Thr289, His291, Phe295, Phe324, Met348, Phe351, Leu353, Ser364 and Gln383 of hAhR was generated using AutoDockTool. Docking was performed with setting the exhaustiveness parameter to 100, in order to improve the sampling effort. The docked pose of the compound with highest binding affinity was selected for further investigation. Ligand interaction diagrams were generated using LigPlot+ software [42]. The visual analysis of dock poses were carried out using PyMOL (The PyMOL Molecular Graphics System, v. 1.7.4, Schrodinger, LLC).

### 4.12. Statistical Analyses

Student *t*-test, one-way analysis of variance (ANOVA) and Dunnett test, were calculated using GraphPad Prism v. 6.0 for Windows (GraphPad Software, La Jolla, CA, USA).

## Supplementary Materials

Supplementary materials can be found at https://www.mdpi.com/1422-0067/21/8/2799/s1. Figures S1: Chemical structures of triptans.

## Abbreviations

CYP1A1	Cytochrome P450 1A1
AhR	Aryl hydrocarbon receptor
ARNT	Aryl hydrocarbon receptor nuclear translocator
TCDD	2,3,7,8-tetrachlorodibenzo-*p*-dioxin
TCDF	2,3,7,8-tetrachlorodibenzofuran
BaP	Benzo[a]pyrene
FICZ	6-Formylindolo[3,2-b]carbazole
IBD	Inflammatory bowel disease
LBD	Ligand-binding domain
DMSO	dimethylsulfoxide
