# Supplementary material for: Antimigraine Drug Avitriptan Is a Ligand and Agonist of Human Aryl Hydrocarbon Receptor that Induces CYP1A1 in Hepatic and Intestinal Cells
Source: Int J Mol Sci. 2020 Apr 17;21(8):2799. doi: 10.3390/ijms21082799 (PMC7216230; doi:10.3390/ijms21082799)
Supplement: Supplementary file 1 [file ijms-21-02799-s001.zip › ijms-762152-supplementary.pdf]

Supplementary materials: Antimigraine drug Avitriptan is a ligand and agonist of human aryl hydrocarbon receptor that induces CYP1A1 in hepatic and intestinal cells

Barbora Vyhlidalova, Kristyna Krasulova, Petra Pecinkova, Karolina Poulikova, Radim Vrzal, Zdenek Andrysik, Aneesh Chandran, Sridhar Mani, Zdenek Dvorak

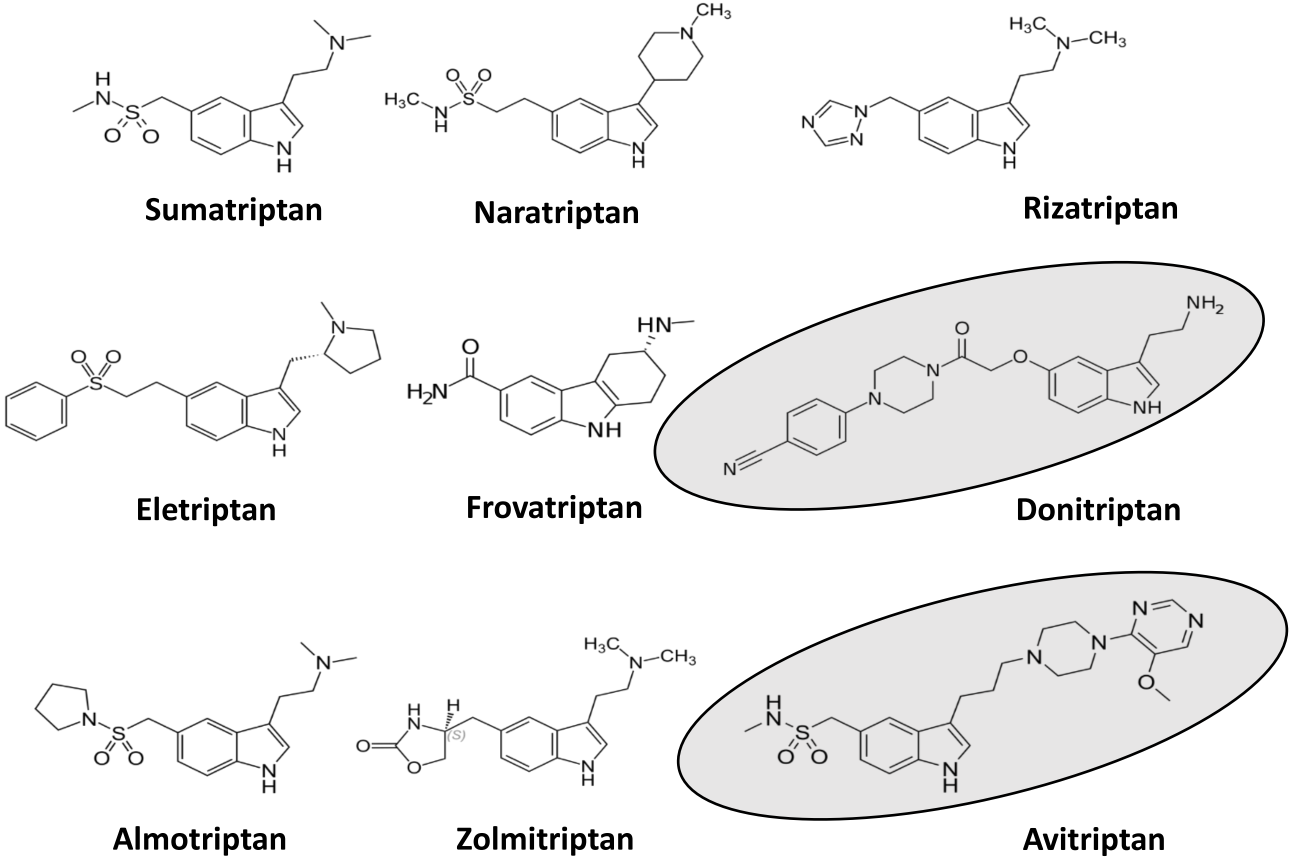

Figure S1. Chemical structures of tested triptans
