# Supplementary material for: Antimigraine Drug Avitriptan Is a Ligand and Agonist of Human Aryl Hydrocarbon Receptor that Induces CYP1A1 in Hepatic and Intestinal Cells
Source: Int J Mol Sci. 2020 Apr 17;21(8):2799. doi: 10.3390/ijms21082799 (PMC7216230; doi:10.3390/ijms21082799)
Supplement: Supplementary file 1 [file ijms-21-02799-s001.zip › ijms-762152-western_blots.pdf]

ORIGINAL WESTERN BLOTS

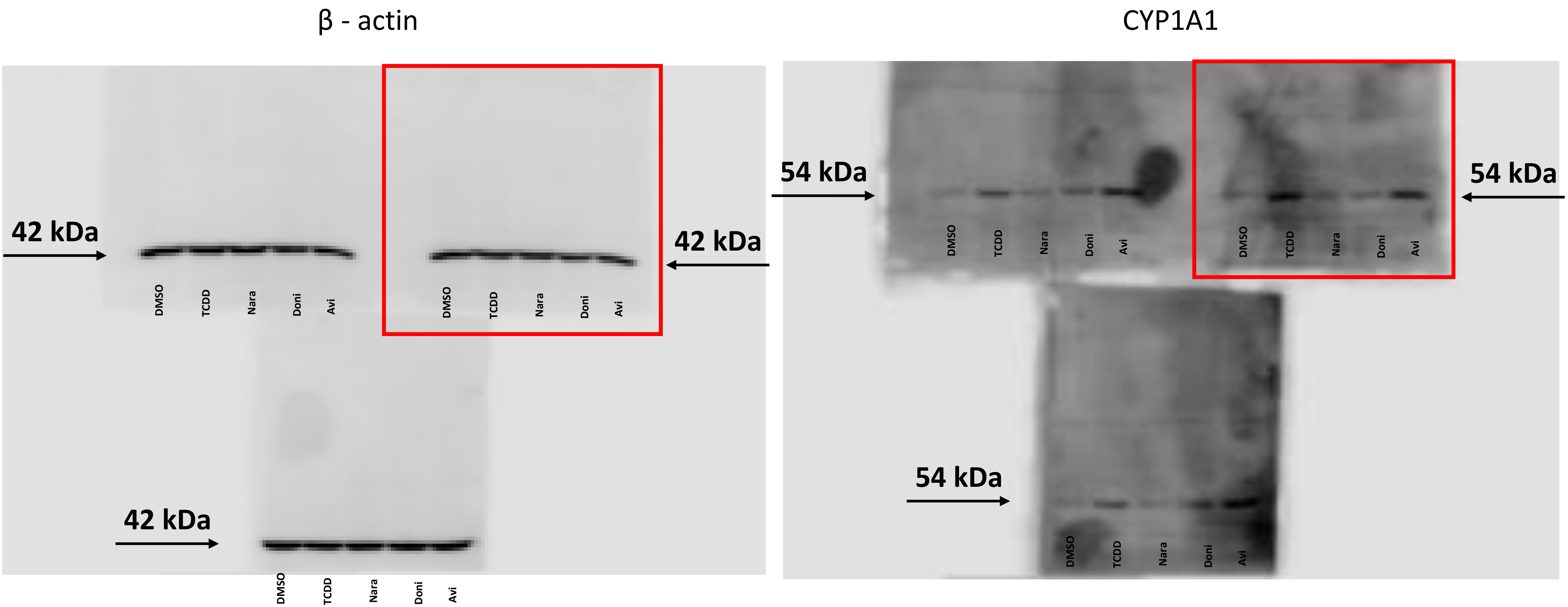

WB labeled with red frame were used for figure 2A

1,5 h

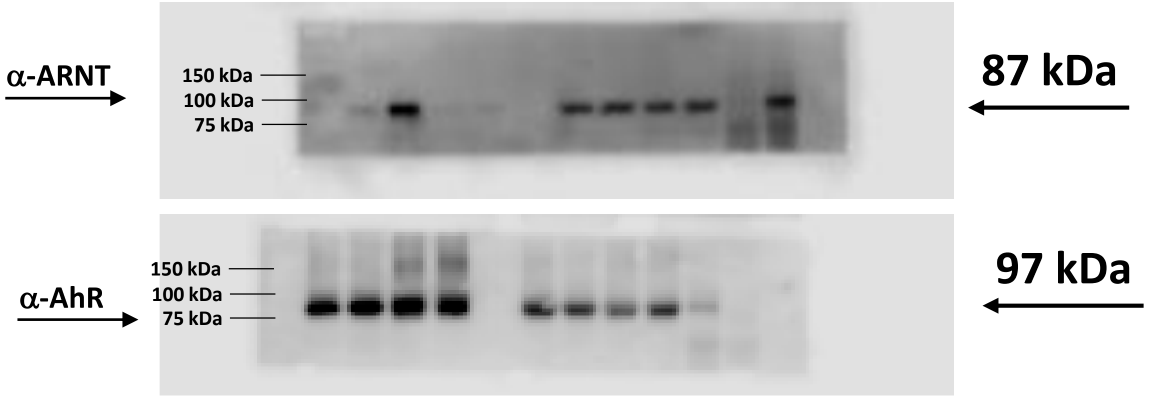

18 h

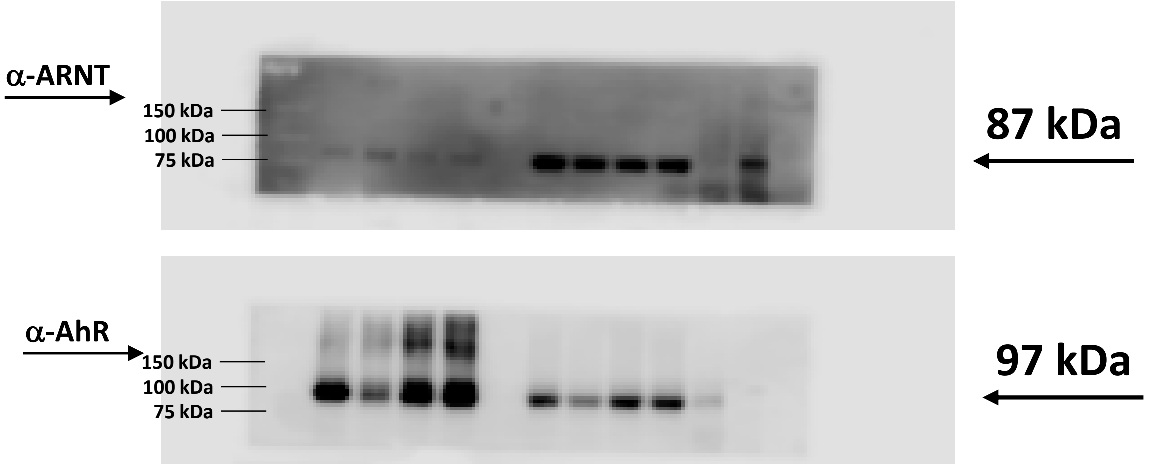

WB were used for figure 6
